# Continuous IV infusion of anakinra

**DOI:** 10.3389/fphar.2023.1162742

**Published:** 2023-05-09

**Authors:** Brittney N. Saunders, Marcela V. Kuijpers, Jason J. Sloan, Elie Gertner

**Affiliations:** ^1^ Regions Hospital, Saint Paul, MN, United States; ^2^ Hennepin Healthcare, Minneapolis, MN, United States

**Keywords:** anakinra, continuous infusion, macrophage activation syndrome, cytokine storm, CAR T-cell

## Abstract

**Objective:** A review of the use of continuous IV infusion of anakinra; a description of the protocol for continuous IV infusion of anakinra in the treatment of cytokine storm developed over the past 4 years at a tertiary level academic medical center in the United States.

**Methods:** We reviewed published reports of continuous IV infusion of anakinra in cytokine storm and summarize this method of treatment in other diseases. As well, over the past 4 years, continuous IV infusions of anakinra were administered at our tertiary level academic medical center in the United States (Regions Hospital, St. Paul, Minnesota) for approximately 400 patient days of treatment primarily for the cytokine storm associated with macrophage activation syndrome (MAS) in adults. This updated protocol is presented. While this a single center protocol, it may serve as an initial guide for further refinement of protocols in MAS and other conditions.

**Conclusion:**Continuous IV infusion of anakinra has advantages over subcutaneous infusions and may be important in controlling severe life-threatening cytokine storm as seen in macrophage activation syndrome. This has the potential to be an important therapy for other syndromes including Cytokine Release Syndrome related to CAR T-cell therapy. Close collaboration between Rheumatology, Pharmacy and Nursing allows this treatment to be delivered rapidly and efficiently.

## Introduction

Cytokine storm syndromes are being recognized more frequently. Whether primarily genetic based or driven by infection, malignancy, or as a complication of autoimmune and autoinflammatory disorders, cytokine storm syndromes are hyperinflammatory conditions associated with marked cytokine release and high mortality (Mehta et al., 2020). MAS/secondary hemophagocytic lymphohistiocytosis (sHLH), is a complication of different rheumatic diseases particularly Still’s Disease, systemic juvenile idiopathic arthritis (sJIA), Adult-Onset Still’s Disease (AOSD) as well as, systemic lupus erythematosus (SLE), systemic vasculitis and Kawasaki Disease among others. Similar syndromes may also result from malignancy and infection. The acute phase of MAS is often associated with extremely high levels of pro-inflammatory cytokines including TNF, IL-6, IL-1β, and IL-18, resulting in a cytokine storm. More recently cytokine release syndrome has become an important complication of CAR T-cell therapies for hematological malignancies (Crayne et al., 2019). In addition to its use in rheumatic disease, anakinra is being utilized in other disease states including pericarditis, gout, and the hyperinflammatory phase associated with infection with SARS-CoV-2 virus.

## Anakinra-subcutaneous and intermittent intravenous administration

Anakinra is a recombinant non-glycosylated form of human interleukin-1 receptor antagonist (IL-1 Ra) used with success in pediatric and adult patients with sJIA, AOSD and subsequently in patients with MAS (Sönmez et al., 2018; Monteagudo et al., 2020). Specific guidelines for drug regimens and duration of treatment are lacking for the latter. It competitively inhibits binding of both IL-1α and IL-1β to the interleukin-1 receptor (IL1-R). The time to achieve maximal plasma concentration after subcutaneous administration at clinically relevant doses is 3–7 h and there is 95% bioavailability (Europa, 2021). The drug initially distributes into a volume corresponding to the plasma volume, followed by distribution into a steady-state volume, similar in magnitude to the extracellular water volume (Europa, 2021). It is renally metabolized and excreted but minimally dialyzable (<2.5%) (Europa, 2021). Since plasma clearance in renal insufficiency is decreased, a dose change should be considered for patients with severe renal insufficiency. The FDA-labeled indications for anakinra are the treatment of Rheumatoid Arthritis, Cryopyrin-Associated Periodic Syndromes (CAPS), and Deficiency of Interleukin-1 Receptor Antagonist (DIRA). However, it is also being used to treat other disease states such as sJIA, AOSD, MAS, Graft *versus* Host Disease, Gout, Kawasaki Disease, Schnitzler Syndrome, Pericarditis, and more recently the hyperinflammatory state of infection with SARS-CoV-2 virus. Adverse effects in rheumatoid arthritis patients (>5%) include injection site reactions, rash, diarrhea, infection, arthralgias, and headache. Rarely bacterial cellulitis, malignant melanoma, cytopenias, anaphylaxis and hypersensitivity reactions can occur. Generally, though, the medication is well tolerated. Neutrophil counts should be assessed prior to initiating treatment, and thereafter depending on the length of treatment.

While most of the early literature focused on the use of subcutaneous anakinra, intravenous administration of anakinra has several advantages. The doses necessary to calm cytokine storm are often high, and multiple subcutaneous injections are painful. Further, in our population of patients with gout who receive anakinra (Thueringer et al., 2015) we have found that subcutaneous injections do not work as well as intravenous therapy in patients with large BMI and edema. This likely represents less rapid systemic cutaneous absorption in obese individuals. Maximal plasma concentration is higher with intravenous administration, and this is extremely important in getting a cytokine storm under control quickly. An excellent review of the safety of IV anakinra is to be found in Mehta et al. (2020).

Even with intermittent intravenous administration of anakinra, we noted that some patients with cytokine storm in MAS experience rapid worsening in the few hours between doses. In these patients, continuous IV anakinra infusion resulted in better outcomes. We have now administered continuous IV anakinra therapy for approximately 400 patient-days over the last 4 years. Given that there is little experience with this therapy in MAS, we review the case reports extant, provide a historical overview of this method of therapy in other disease states, and describe the Regions Hospital protocol. Regions Hospital is a tertiary care academic medical center located in St. Paul, Minnesota and serves as a referral center for complex rheumatological diseases [The Institutional Review Board and the HealthPartners Institute Research Subjects Protection Program did not consider the development and subsequent publication of this single center protocol as meeting the definition of human subjects research under 45 CFR Part 46].

## Continuous IV infusion of anakinra

### Other disease states

Continuous IV infusion of anakinra was first reported in the treatment of sepsis and acute graft vs. host disease (Antin et al., 1994; Fisher et al., 1994). Patients with sepsis were given a loading dose of anakinra 100 mg followed by a continuous 72-h infusion. Anakinra did not meet the primary endpoint of improved 28-day survival compared to placebo, but secondary and retrospective analyses of efficacy suggested that treatment with anakinra led to improvement in survival in a subset of patients with features of MAS (Shakoory et al., 2016).

Inflammatory cytokines, such as IL-1 may be important in acute graft *versus* host disease. An open-label phase I/II trial explored the safety of anakinra in patients with steroid resistant acute graft *versus* host disease (Antin et al., 1994). Anakinra was given by continuous intravenous infusion in doses from 400 to 3200 mg/day for 7 days. There was some improvement, and except for a reversible rise of liver transaminases seen in two patients, appeared safe. Patients with subarachnoid hemorrhage (Singh et al., 2014) or stroke (Emsley et al., 2005) have also been treated with continuous IV anakinra infusions.

### Macrophage activation syndrome

While similar in their inflammatory pathophysiology, primary HLH, a familial or genetic syndrome, and sHLH, are recognized as separate subsets of the disease spectrum (Monteagudo et al., 2020). MAS is a subset of HLH often associated with rheumatologic diseases including, sJIA, SLE, and AOSD as well as vasculitis and Kawasaki disease. MAS associated with underlying rheumatic conditions can also be triggered by infections, such as Epstein‐Barr virus and varicella zoster virus (Monteagudo et al., 2020). The clinical manifestations include high, persisting fevers, malaise, and multisystem organ dysfunction. Markedly elevated transaminases, triglycerides, lactate dehydrogenase, and ferritin levels (the latter to extremely high levels) are characteristic. Peripheral blood cytopenias, hypofibrinogenemia, elevated soluble CD25 (soluble IL-2 receptor alpha) levels as well as hemophagocytosis in bone marrow, liver or spleen may be present. The diagnosis of MAS is often delayed and not recognized particularly in adults resulting in high mortality. Early diagnosis and rapid initiation of therapies aimed at reducing systemic inflammation are critical to the management of MAS (Monteagudo et al., 2020).

Continuous IV anakinra infusions of up to 2400 mg/d were used by Monteagudo et al. (Monteagudo et al., 2020) to treat severely ill adult patients with MAS/sHLH with 2 mg/kg/hour as the maximal dose. In children, three critically ill children (ages 9, 11, and 17) with severe sHLH and rapidly progressive multiorgan dysfunction were successfully treated with continuous IV anakinra infusions. The series highlighted the safety and efficacy of IV anakinra for life-threatening sHLH. Doses ranged from 11 mg/kg/day to 12 mg/kg/day to 2 mg/kg/hour (Charlesworth et al., 2021). Kavirayani et al. described a Lazarus effect of high dose IV anakinra treatment for severe non-familial central nervous system-HLH in a 9-year-old female patient. When the dose was increased to 2 mg/kg/hour noticeable improvement occurred (Kavirayani et al., 2020).

There are currently no reports on the use of continuous IV anakinra for the spectrum of cytokine release syndrome associated with CAR T-cell therapy for the treatment of patients with relapsed or refractory large B cell lymphoma although subcutaneous anakinra is being studied.

Given our experience with continuous IV anakinra infusion we present the Regions Hospital protocol that we have used over the past 4 years in Appendix A. There is very little published data on stability. Charlesworth et al. (2021) studied continuous infusions of anakinra and changed syringes every 6–8 h. In our experience, we also do bag changes every 6–8 h-but insist that there be absolutely no delay after that.

To summarize: We initiate 1) higher doses of anakinra when a diagnosis is made (often 400–600 mg/day [i.e., approximately 0.25–0.5 mg/kg/hour in a 70 kg patient]) 2) escalate the dose rapidly if clinical signs and laboratory studies suggest ongoing disease and 3) insist on no interruptions during the continuous IV therapy.

## Conclusion

Continuous IV infusion of anakinra represents an important therapeutic option for patients with cytokine storm due to different hyperinflammatory states. It may have further applications for cytokine release syndrome secondary to complications from CAR T-cell therapy. Early initiation and rapid escalation of therapy can lead to satisfactory outcomes. Coordination between the clinician, nurses and pharmacy teams will insure appropriate, timely use with good responses.

## Data Availability

The original contributions presented in the study are included in the article/Supplementary Material, further inquiries can be directed to the corresponding authors.

## APPENDIX A

Regions Hospital Continuous IV Infusion of Anakinra Protocol.

Dose will be ordered as mg/day. Dosing ranges up to 2 mg/kg/day IV based on clinical and lab monitoring.

IV pharmacy will take total daily dose ÷ 3 and make every 8-h bags.

Anakinra should not be sent via pneumatic tube system and is hand delivered by pharmacy technicians.

Anakinra should be compounded to an IV bag concentration between 1–5 mg/mL. Regions utilizes a minimum bag size of 50 mL to reduce amount of lost drug with priming bags.

Anakinra needs to be protected from light. Every dose is sent in a ‘protect from light’ bag AND with 3 feet of “protect from light” amber tubing for the IV line.

Administration instructions on every IV bag include:

“This product needs to be protected from light, including both the final container bag and the tubing. Pharmacy will send each bag in a light protected outer bag and amber tube covering with the first dose and then on request to nursing floors when needed.”

Always round the dose to the nearest 100 mg–change bags to q6h if this facilitates rounding.

Example: Rheumatology orders anakinra continuous IV infusion at 300 mg/day. To build this order, we put anakinra 100 mg in 100 ml NS and set the rate at 12.5 ml/h (each bag lasts 8 h, and patient receives three bags/day for a total of 300 mg). The next day, Rheumatology increases the dose to 600 mg/day. We change the order to anakinra 200 mg in 100 ml NS, leaving the rate at 12.5 ml/h.

Ordering Process

1. Regions Hospital utilizes a health system designed Epic order created with the Epic team and pharmacy P&T committee.
2. Select “anakinra IV infusion” in orders tab.
3. Since this medication is restricted formulary enter the designated approving service.
4. Type indication for IV anakinra infusion
5. Enter duration of infusion (example: 24-h continuous infusion)
6. Enter dose
7. Select desired bag size (50, 100, and 250 ml)

Pharmacy Verification Process

1. Order will come across with anakinra continuous infusion with directions indicating total daily dose and approving restricted formulary service.
2. Pharmacy will divide doses into an even interval and dose rounded to the nearest 100 mg to not waste syringes.
3. This is a pharmacy-built order and will require the selection of dose, frequency, size of bag and rate to run at.
4. The order itself will still say continuous infusion; however, nursing follows the rate on the order and requests new bags in ample time before current bag completed.

Compounding Process

This is assuming all other legal and facility required sterile product compounding tasks are completed.

1. Obtain anakinra syringes from the refrigerator and NS bag from stock room (this may be 50 or 100 ml bag).
2. Wipe down injection port of NS bag and inject instructed number of anakinra syringes into port.
3. Cover in “protect from light” bag, perform other required compounding activities, and dispense at least 2–3 feet of ‘protect from light’ cover for tubing.
4. Anakinra bags are made within an hour of administration to limit drug wasting.

Administration Process

1. Bag must be hung with protect from light covering including on tubing.
2. Anakinra IV infusion was added as a pump setting on smart pump system.
3. Bags are administered as continuous infusion over 8 h.
4. Nursing is responsible for reaching out to pharmacy for next dose.
